# High-Dose Inhaled Nitric Oxide as Adjunct Therapy in Cystic Fibrosis Targeting *Burkholderia multivorans*

**DOI:** 10.1155/2020/1536714

**Published:** 2020-06-24

**Authors:** Bethany L. Bartley, Kelly J. Gardner, Stefano Spina, Bryan P. Hurley, David Campeau, Lorenzo Berra, Lael M. Yonker, Ryan W. Carroll

**Affiliations:** ^1^Department of Pediatric Pulmonology, Massachusetts General Hospital, 55 Fruit Street, Boston, MA 02114, USA; ^2^The Mucosal Immunology and Biology Research Center, Massachusetts General Hospital East, 16^th^ Street, Charlestown, MA 02129, USA; ^3^Department of Anesthesia, Massachusetts General Hospital, 55 Fruit Street, Boston, MA 02114, USA; ^4^Harvard Medical School, 25 Shattuck Street, Boston, MA 02115, USA; ^5^Department of Pediatric Critical Care Medicine, Massachusetts General Hospital, 55 Fruit Street, Boston, MA 02114, USA

## Abstract

**Background:**

Individuals with cystic fibrosis (CF) have persistent lung infections, necessitating the frequent use of antibiotics for pulmonary exacerbations. Some respiratory pathogens have intrinsic resistance to the currently available antibiotics, and any pathogen may acquire resistance over time, posing a challenge to CF care. Gaseous nitric oxide has been shown to have antimicrobial activity against a wide variety of microorganisms, including common CF pathogens, and offers a potential inhaled antimicrobial therapy. *Case Presentation*. Here, we present the case of a 16-year-old female with CF who experienced a precipitous decline in lung function over the prior year in conjunction with worsening antibiotic resistance of her primary pathogen, *Burkholderia multivorans*. She received 46 intermittent inhalations of 160 parts-per-million nitric oxide over a 28-day period. The gas was administered via a mechanical ventilator fitted with nitrogen dioxide scavenging chambers.

**Conclusions:**

High-dose inhaled nitric oxide was safe, well tolerated, and showed clinical benefit in an adolescent with cystic fibrosis and pulmonary colonization with *Burkholderia multivorans*.

## 1. Introduction

Cystic fibrosis (CF) is a genetic disease characterized by recurrent lung infection and inflammation, which leads to progressive lung damage and respiratory insufficiency. *Burkholderia multivorans* (*B*. *multivorans*) belongs to a group of Gram-negative bacteria known as the *Burkholderia cepacia* (*B*. *cepacia*) complex. Bacteria from this group infect the lungs of only 2.4% of individuals with CF [1], but have inherent resistance to many common antibiotics, and colonization often leads to a decline in lung function [2].

Nitric oxide (NO) plays a key role in host defense mechanisms in the lung and endogenous production of NO is decreased in CF airways [3]. The US Food and Drug Administration (FDA) initially approved inhaled NO as treatment for pulmonary hypertension in newborns using 20 parts-per-million (ppm) [4]. Off-label applications have been explored, and intermittent high-dose concentrations of 160 ppm have been shown to have antimicrobial activity against CF pathogens *in vitro* [5] and in an animal model [6]. Inhaled intermittent dosing of 160 ppm NO was found to be safe and well tolerated in healthy adult volunteers [7], as well as in individuals with CF and respiratory colonization with common CF pathogens including *Pseudomonas aeruginosa* (*P*. *aeruginosa*) [8] and *Mycobacterium abscessus* [9]. A phase II clinical study is currently underway to evaluate the safety and efficacy of high-dose inhaled NO in adults with CF and chronic respiratory colonization with *P*. *aeruginosa*, *Staphylococcus aureus*, or *Stenotrophomonas maltophilia* (ClinicalTrials.gov, Identifier: NCT02498535). Previous to this report, the use of high-dose inhaled NO in an individual with CF and pulmonary colonization with *B*. *multivorans* has not been described.

## 2. Case Presentation

**Figure 1:**
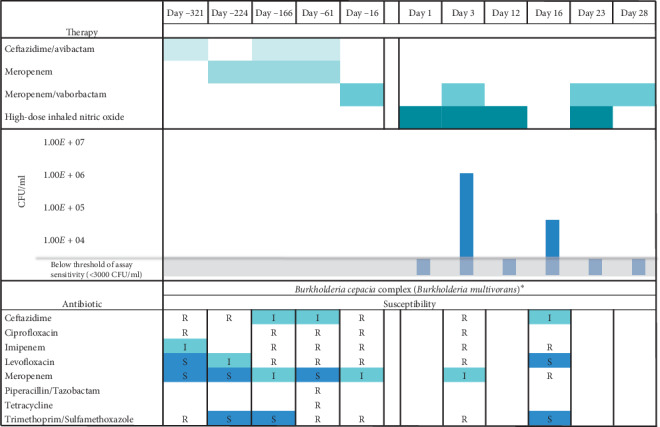
Timeline of antibiotic therapies, antibiotic susceptibility pattern of the primary pathogen (*Burkholderia cepacia* complex (*Burkholderia multivorans*)^*∗*^), and colony forming units (CFU) of this pathogen relative to the initiation of high-dose inhaled nitric oxide therapy on day 1. The antibiotic susceptibility data are reported as susceptible S, intermediate I, or resistant R. ^*∗*^Due to limitations in commercial identification systems to reliably distinguish among species of the *Burkholderia cepacia* complex, *Burkholderia multivorans* identification was reported as the *Burkholderia cepacia* complex by our clinical microbiology laboratory. The patient's isolates had been confirmed as *Burkholderia multivorans* by the *Burkholderia cepacia* Research Laboratory and Repository (Ann Arbor, Michigan, USA) within the prior year.

The patient is a 16-year-old female with CF (genotype: F508del/W1282X), CF-related diabetes requiring insulin, and CF-related liver disease after liver transplant, who experienced a precipitous decline in lung function over the prior year, requiring frequent- near monthly -hospital admissions for intravenous antibiotics targeting her primary respiratory pathogen, *B*. *multivorans*. The patient acquired this pathogen in her sputum six years before, and over time, it developed increasing antibiotic resistance (Figure 1). Notably, the patient required long-term immunosuppressive therapy following liver transplant 21 months before. Her frequency of hospital admission for pulmonary exacerbations remained unchanged for the first year after liver transplant but doubled in the succeeding 9 months. Her pulmonary exacerbations would only briefly respond to a single antibiotic, intravenous meropenem-vaborbactam. Furthermore, the patient's weight remained stagnant at the 10^th^ percentile.

Female sex, body mass index ≤18 kg/m^2^, CF-related diabetes requiring insulin, *B*. *cepacia* complex infection, and frequent pulmonary exacerbations have all been identified as risk factors for mortality in individuals with CF and low lung function [10]. Cystic fibrosis transmembrane conductance regulator (CFTR) modulators have been shown to increase lung function and decrease rates of pulmonary exacerbation in individuals with CF [11, 12]; however, the patient's genotype and history of liver transplantation precluded her from qualifying for any CFTR modulators available at the time or therapeutic trials, respectively. Lung transplantation was discussed though the patient's colonizing pathogen and adolescent age are controversially associated with poorer outcomes [13, 14].

High-dose inhaled NO administration via a mechanical ventilator fitted with scavenging chambers was reviewed and approved under an institutional process called “Innovative Diagnostics and Therapeutics,” by which independent hospital leadership evaluate novel approaches to the care of individual patients. The patient assented, and her parents provided consent for this therapy.

### 2.1. Treatment and Outcome

**Figure 2:**
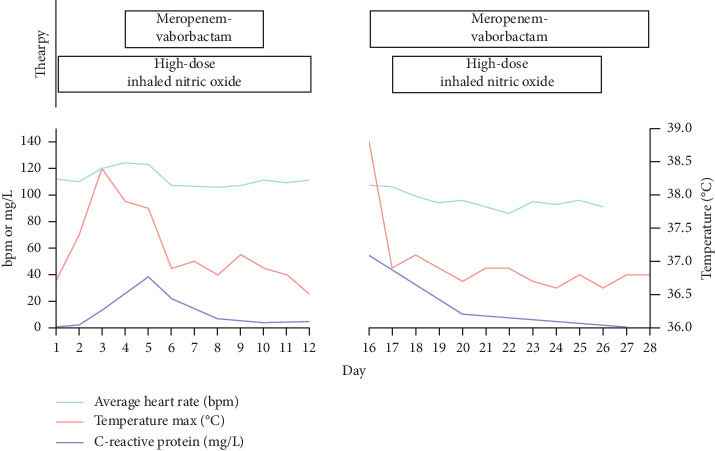
Relationship between therapies, vital signs, and inflammatory markers in an adolescent with CF, treated with intravenous antibiotics and high-dose inhaled nitric oxide. The first and second hospital admissions are shown on the left and right panels of the figure, respectively. Duration of therapy with either intravenous antibiotics or inhaled nitric oxide is shown at the top of the figure.

The patient received a total of 46 intermittent inhalations of 160 ppm NO over a 28-day period during two separate hospital admissions: days 1–12 and days 16–28 (Figure 2). During the first admission, inhaled NO was given over 30-minute intervals up to three times daily (27 total doses). In the second admission, the treatment interval was gradually increased to 60 minutes, two times daily (19 total doses), starting with 30-minute inhalations up to three times daily (9 doses), transitioned to 45-minute inhalations twice daily (2 doses), and ultimately 60-minute inhalations twice daily (8 doses). She concurrently received intravenous meropenem-vaborbactam (dose: 4 grams every 8 hours) on days 4–10 and 16–28.

**Figure 3:**
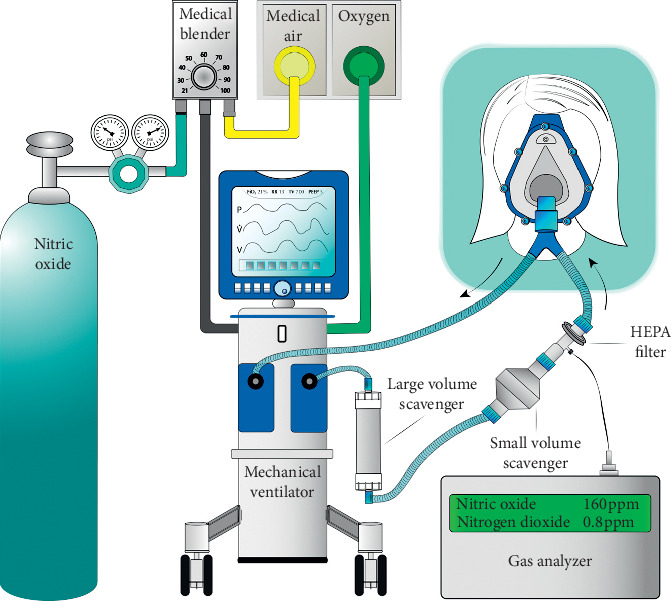
Schematic representation of the inhaled nitric oxide delivery system. The nitric oxide gas source was a 850 ppm nitric oxide in nitrogen tank. This gas was mixed with medical air in a gas blender, and the resulting gas mixture was blended with oxygen in the mechanical ventilator. The blender and FiO_2_ dial were both adjusted to achieve the targeted nitric oxide and oxygen concentrations. Large and small nitrogen dioxide scavengers were placed in series along the inspiratory limb. The gas mixture was delivered to the patient via a sealed facemask. Delivered gas was sampled just proximal to the patient, and nitric oxide and nitrogen dioxide concentrations were analyzed with a portable gas analyzer.

The NO gas source was commercially available as 850 ppm nitric oxide in nitrogen tanks that meet the Environmental Protection Agency (EPA) traceability protocol requirements (Airgas Inc., Radnor Township, Pennsylvania, USA). Oxygen, medical air, and NO were blended and introduced through a ventilator (Puritan Bennett 980 Series, Medtronic, Minneapolis, Minnesota, USA) (Figure 3). A target level of 160 ppm inhaled NO and 0.21 FiO_2_ were delivered via a tight-fitting noninvasive mask using a pressure support mode of 2 cmH_2_O driving pressure over a positive end-expiratory pressure of 5 cmH_2_O. Because the NO source of 850 ppm is mixed with nitrogen, the ventilator was set to a range of 0.25-0.26 FiO_2_, in order to deliver an FiO_2_ of 0.21 to the patient.

Nitric oxide and nitrogen dioxide (NO_2_) analyzers were attached to a sampling port proximal to the facemask and provided real-time measurements. Nitrogen dioxide, a potentially harmful gas that develops when oxygen and NO are mixed, was scavenged with calcium hydroxide chambers (Spherasorb™, Intersurgical Ltd, Berkshire, UK), and levels remained less than 1.5 ppm in the circuit. Ambient NO and NO_2_ levels were intermittently measured in the patient's room and consistently remained below EPA recommendations [15] at less than 4 parts-per-billion (ppb) and 12 ppb, respectively.

**Figure 4:**
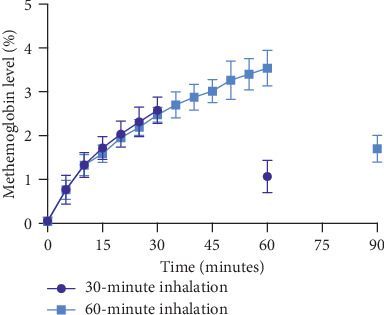
Methemoglobin levels (mean ± standard deviation) from data recorded at 5-minute intervals during 30-minute and 60-minute inhalations of high-dose nitric oxide. Curves demonstrate a reproducible and expected rise in methemoglobin during therapy and reduction 30 minutes after cessation (note: data from 45-minute inhalations were omitted for clarity, but follow a similar rise, peak, and decrease 30 minutes post-inhalation).

There were no serious adverse events, and inhalations were well tolerated. Echocardiograms were performed before, during, and after the first inhalation and demonstrated normal estimated pulmonary pressures. Heart rate, respiratory rate, oxygen saturation, and blood pressure were recorded at 5-minute intervals throughout inhalations and 30 minutes after cessation. The patient did not experience any hemodynamic instability or significant hypoxia with treatments. Nitric oxide oxidizes *ferrous* (Fe^2+^) hemoglobin into the *ferric* state (Fe^3+^), known as methemoglobin. Methemoglobin levels were continuously monitored during inhalations using a peripheral pulse co-oximeter (Masimo Rainbow Set^TM^ Technology, Irvine, CA) and never surpassed our safety threshold of 5%. Specifically, at the end of the 30-minute and 60-minute inhalations, the highest recorded methemoglobin levels were 3.1% and 4.1%, respectively, and returned to baseline prior to subsequent treatments (Figure 4). Additionally, the patient received 125 mg of ascorbic acid by mouth daily (on treatment days) to enhance methemoglobin reductase activity [16].

Sputum was collected prior to (days 1 and 16), midcycle (days 3 and 23), and on completion (days 12 and 28) of each NO cycle (Figure 1). Colony forming units (CFU) were calculated using sputum solubilized with Sputolysin^®^ (MilleporeSigma) and plated on *Burkholderia cepacia* isolation agar. On days 1, 12, 23, and 28, *B*. *multivorans* grew from the patient's sputum, but the CFU count was low (Figure 1). Early in therapy (day 3), the sputum culture yielded highly antibiotic-resistant *B*. *multivorans*, consistent with months before; however, by the end of the first week of therapy (day 16), isolated *B*. *multivorans* demonstrated susceptibility to trimethoprim-sulfamethoxazole and levofloxacin and intermediate susceptibility to ceftazidime, a susceptibility pattern not seen for months.

Fever, white blood cell count, and C-reactive protein all decreased with therapy (Figure 2). The patient never manifested bacteremia or hemodynamic instability suggestive of *cepacia* syndrome—a dreaded complication of *B*. *cepacia* complex infection primarily linked to *Burkholderia cenocepacia* though also associated with *B*. *multivorans* [2]. The patient's international normalized ratio (INR) remained consistently below 1.2, and she never demonstrated evidence of platelet dysfunction, hemoptysis, or other bleeding. Her lung function improved from her baseline. Specifically, on day 1—before starting high-dose inhaled NO therapy and after recently completing a 16-day course of intravenous meropenem-vaborbactam two days before—the patient's forced expiratory volume in 1 second (FEV_1_) and forced vital capacity (FVC) were 1.60 L (49% predicted) and 2.05 L (56% predicted), respectively. Both measurements improved at the end of the second admission to FEV_1_ 1.72 L (52% predicted) and FVC 2.36 L (64% predicted). The patient's baseline weight in the prior 6 months remained at the 10^th^ percentile, and her BMI was consistently below 18 kg/m^2^. Her weight at the start of inhaled NO therapy was 48.4 kg (22^nd^ percentile). It rose to a maximum of 50.3 kg (30^th^ percentile) on day 16 but decreased to 48.9 kg (24^th^ percentile) by day 24.

## 3. Discussion

Here, we describe the inpatient use and tolerability of high-dose inhaled NO as an adjunct anti-infective targeting highly resistant *B*. *multivorans* in an adolescent with CF. Consistent with previous reports [7–9], there were no significant adverse events. An increase in the patient's pulmonary function and weight was observed relative to baseline. Additionally, the antibiotic resistance pattern of *B*. *multivorans* improved, demonstrating susceptibility to trimethoprim-sulfamethoxazole and levofloxacin, where resistance to these antibiotics had previously been persistent.

The change in the antibiotic resistance pattern after inhaled NO therapy is promising though challenging to interpret. Studies on the bactericidal mechanism of high-dose NO in *Burkholderia* bacteria are limited though given its proposed indiscriminate mechanism of membrane protein modification, DNA damage, and nitrosative and oxidative stress [17, 18], and it is possible that high-dose NO exerted direct toxic effects on the bacteria contributing to a reduction in CFU and killing of more resistant isolates. An alternative hypothesis may be biofilm dispersal. Nitric oxide is thought to mediate dispersal by diffusing into biofilms and upregulating bacterial phosphodiesterases which inhibit or degrade the second messenger and biofilm regulator, cyclic-di-guanosine monophosphate (c-di-GMP) [17, 19]. Low-dose NO has been shown to induce biofilm dispersal of *P*. *aeruginosa* in an *ex vivo* model [17], and a recent study demonstrated the antibiofilm properties of NO donors with common CF pathogens, including the *B*. *cepacia* complex, *in vitro* [20]. In this clinical case, it is possible that biofilms sheltered more antibiotic sensitive isolates of *B*. *multivorans* that were released into the planktonic state, and thus more susceptible to antibiotic-mediated killing after the initiation of high-dose inhaled NO.

## 4. Conclusion

Inhaled high-dose NO therapy, utilized as an anti-infective adjunct therapy, improved pathogen antibiotic resistance patterns and clinical outcomes in our patient. Mechanistic studies *in vitro* and larger randomized controlled clinical trials are needed to better understand the bactericidal efficacy and biofilm dispersal properties of high-dose NO on *B*. *multivorans*. To our knowledge, this is the first report of high-dose inhaled NO therapy in an individual with CF and pulmonary colonization with *B*. *multivorans*. In follow-up, the patient continues to require frequent courses of antibiotics for pulmonary exacerbations though much less frequent inpatient admissions. During inpatient stays, we continue to offer high-dose inhaled NO therapy as an adjunct therapy to intravenous antibiotics. The patient's genotype qualifies for the newly available, highly effective, triple combination CFTR modulator, elexacaftor-ivacaftor-tezacaftor (Vertex Pharmaceuticals) [21], which has improved her lung function but has not yet altered her sputum microbiota. Recalcitrant infection with multidrug-resistant pathogens will likely continue to be a major problem in CF despite the advent of CFTR modulators [22, 23], and high-dose inhaled NO may help those who continue to struggle with difficult to treat respiratory pathogens.

## Data Availability

The data collected and analyzed for this single-patient study are available from the corresponding author upon reasonable request.

## References

[1] Marshall B., Faro A., Fink A. (2018). *Cystic fibrosis foundation patient registry, 2017 Annual Data Report*.

[2] Gilligan P. H. (2014). Infections in patients with cystic fibrosis. *Clinics in Laboratory Medicine*.

[3] Grasemann H., Al-Saleh S., Scott J. A. (2011). Asymmetric dimethylarginine contributes to airway nitric oxide deficiency in patients with cystic fibrosis. *American Journal of Respiratory and Critical Care Medicine*.

[4] US Food and Drug Administration (2009). INOmax (nitric oxide) for inhalation. https://www.accessdata.fda.gov/drugsatfda_docs/label/2009/020845s009lbl.pdf.

[5] Miller C., McMullin B., Ghaffari A. (2009). Gaseous nitric oxide bactericidal activity retained during intermittent high-dose short duration exposure. *Nitric Oxide*.

[6] Miller C. C., Hergott C. A., Rohan M., Arsenault-Mehta K., Döring G., Mehta S. (2013). Inhaled nitric oxide decreases the bacterial load in a rat model of *Pseudomonas aeruginosa* pneumonia. *Journal of Cystic Fibrosis*.

[7] Miller C., Miller M., McMullin B. (2012). A phase I clinical study of inhaled nitric oxide in healthy adults. *Journal of Cystic Fibrosis*.

[8] Deppisch C., Herrmann G., Graepler-Mainka U. (2016). Gaseous nitric oxide to treat antibiotic resistant bacterial and fungal lung infections in patients with cystic fibrosis: a phase I clinical study. *Infection*.

[9] Bentur L., Gur M., Ashkenazi M. (2020). Pilot study to test inhaled nitric oxide in cystic fibrosis patients with refractory *Mycobacterium abscessus* lung infection. *Journal of Cystic Fibrosis*.

[10] Ramos K. J., Quon B. S., Heltshe S. L. (2017). Heterogeneity in survival in adult patients with cystic fibrosis with FEV <30% of predicted in the United States. *Chest*.

[11] McKone E. F., Borowitz D., Drevinek P. (2014). Long-term safety and efficacy of ivacaftor in patients with cystic fibrosis who have the Gly551Asp-CFTR mutation: a phase 3, open-label extension study (PERSIST). *The Lancet Respiratory Medicine*.

[12] Taylor-Cousar J. L., Munck A., McKone E. F. (2017). Tezacaftor-Ivacaftor in patients with cystic fibrosis homozygous for Phe508del. *New England Journal of Medicine*.

[13] Morrell M. R., Pilewski J. M. (2016). Lung transplantation for cystic fibrosis. *Clinics in Chest Medicine*.

[14] Hayes D., Cherikh W. S., Chambers D. C. (2019). The International Thoracic Organ Transplant Registry of the International Society for Heart and Lung Transplantation: twenty-second pediatric lung and heart-lung transplantation report—2019; focus theme: donor and recipient size match. *The Journal of Heart and Lung Transplantation*.

[15] The United States Environmental Protection Agency (2012). Nitric oxides AEGL technical support document. *Acute Exposure Guideline Levels for Selected Airborne Chemicals*.

[16] Mwanga-Amumpaire J., Carroll R. W., Baudin E. (2015). Inhaled nitric oxide as an adjunctive treatment for cerebral malaria in children: a phase II randomized open-label clinical trial. *Open Forum Infectious Diseases*.

[17] Howlin R. P., Cathie K., Hall-Stoodley L. (2017). Low-dose nitric oxide as targeted anti-biofilm adjunctive therapy to treat chronic *Pseudomonas aeruginosa* infection in cystic fibrosis. *Molecular Therapy*.

[18] Möller M. N., Li Q., Lancaster J. R., Denicola A. (2007). Acceleration of nitric oxide autoxidation and nitrosation by membranes. *IUBMB Life*.

[19] Barraud N., Schleheck D., Klebensberger J. (2009). Nitric oxide signaling in *Pseudomonas aeruginosa* biofilms mediates phosphodiesterase activity, decreased cyclic Di-GMP levels, and enhanced dispersal. *Journal of Bacteriology*.

[20] Ahonen M. J., Dorrier J. M., Schoenfisch M. H. (2019). Antibiofilm efficiency of nitric oxide-releasing alginates against cystic fibrosis bacterial pathogens. *ACS Infectious Diseases*.

[21] Middleton P. G., Mall M. A., Dřevínek P. (2019). Elexacaftor-tezacaftor-ivacaftor for cystic fibrosis with a single Phe508del Allele. *New England Journal of Medicine*.

[22] Frost F. J., Nazareth D. S., Charman S. C., Winstanley C., Walshaw M. J. (2019). Ivacaftor is associated with reduced lung infection by key cystic fibrosis pathogens, a cohort study using National Registry Data. *Annals of the American Thoracic Society*.

[23] Heltshe S. L., Mayer-Hamblett N., Burns J. L. (2015). *Pseudomonas aeruginosa* in cystic fibrosis patients with G551D-CFTR treated with ivacaftor. *Clinical Infectious Diseases*.

